# Prevalence of undernutrition and potential risk factors among children below five years of age in Somali region, Ethiopia: evidence from 2016 Ethiopian demographic and health survey

**DOI:** 10.1186/s40795-021-00460-0

**Published:** 2021-10-07

**Authors:** Damitie Kebede, Alebel Aynalem

**Affiliations:** 1 College of Agriculture and Environmental Sciences, Bahir Dar University, P O Box, 5501, Bahir Dar, Ethiopia; 2 Institute of Disaster Risk Management and Food Security Studies, Bahir Dar University, P O Box 5501, Bahir Dar, Ethiopia

**Keywords:** Young children, Underweight, Wasting, Stunting, Ethiopia

## Abstract

**Background:**

Childhood under-nutrition is far-reaching in low and middle-income nations. Undernutrition is one of the major open wellbeing concerns among newborn children and youthful children in Ethiopia. The present study aimed to explore the potential risk factors of undernutrition among children under 5 years of age in Somali Region, Ethiopia.

**Methods:**

The data for this study was extricated from the Ethiopian Demographic and Health Survey (EDHS) 2016. The data collected from 1339 children born 5 years before was considered within the analysis. A multivariable binary logistic regression analysis was utilized at a 5% level of significance to decide the individual and community-level variables related to childhood malnutrition.

**Results:**

The prevalence of stunting, underweight and wasting were 27.4, 28.7 and 22.7%, respectively. About 16.1% of children were both stunted and underweight; the extent of both being underweight and wasted was 11.7%, the prevalence of both stunted and wasted children was 5.5%, and all three malnutrition conditions were 4.7% children. Among the variables considered in this study, age of the child in months, type of birth, anemia level, size of child at birth, sex of the child, mothers’ BMI and sources of drinking water were significantly related to stunting, underweight and wasting in Somali Region.

**Conclusions:**

The prevalence of stunting, underweight and wasting was relatively high. Undernutrition is one of the major open wellbeing concerns among children in Somali region. The impact of these variables ought to be considered to develop strategies for decreasing the lack of healthy sustenance due to undernutrition in the study areas. Hence, intercession should be centered on making strides for the under-nutrition determinant variables of the children to be solid, to improve the child’s wholesome status, and decrease child mortality quickly.

## Background

Adequate nutrition is essential in early childhood to ensure healthy growth, proper organ formation and function, a strong immune system, and neurological and cognitive development. Economic growth and human development require well-nourished populations who can learn new skills, think critically and contribute to their communities. Child malnutrition impacts cognitive function and contributes to poverty by impeding individuals’ ability to lead productive lives. In addition, it is estimated that more than one-third of under-five deaths are attributable to undernutrition [1, 2].

Nutrition has increasingly been recognized as a basic pillar for social and economic development. The reduction of infant and young child malnutrition is essential to the achievement of the Millennium Development Goals (MDGs) particularly those related to the eradication of extreme poverty and hunger (MDG 1) and child survival (MDG 4). Improving children’s nutrition requires effective and sustained multi-sectoral nutrition programming over the long term, and many countries are moving in the right direction [3].

Globally, an estimated 165 million children under 5 years of age, or 26%, were stunted, 101 million children under 5 years of age, or 16%, were underweight and 52 million children under 5 years of age, or 8%, were wasted. High prevalence levels of stunting among children under 5 years of age in Africa (36% in 2011) and Asia (27% in 2011) remain a public health problem, one which often goes unrecognized. More than 90% of the world’s stunted children live in Africa and Asia. Although the prevalence of stunting, underweight and wasting among children under 5 years of age worldwide has decreased since 1990, overall progress is insufficient and millions of children remain at risk [3].

Childhood under-nutrition is broad in low and middle-income nations. In these nations, it is an imperative and backhanded cause of child mortality. Around the world, stunting and wasting besides intrauterine development limitations are mindful of about 2.1 million deaths in under-five children that contain 21% of all deaths [2].

Undernutrition in children occurs due to the interplay of several factors, which include variables related to the maternal age, maternal education, poor feeding practice, maternal nutritional status, parity and multiple births, sex of the child, illness, birth interval and immunization status, poor wealth status, large families, water and sanitation, place of residence, and other factors relating to health services utilization [4–7]. Child under-nutrition and mortality rates are sensible reactions to both wellbeing programs and financial conditions such as pay, unemployment, and lack of education [8]. In Ethiopia, the predominance of under-nutrition is high [9–13] which ranges from 14.6% in Addis Ababa to 46.3% in Amhara region for stunting, 3.5% in Addis Ababa to 22.5% in Somalia region for wasting and 5% in Addis Ababa to 35.5% in Somali region for underweight [14].

Although a few studies have been carried out on the identification of factors that are associated with children under 5 years old of undernutrition in the Somali region, none of them uses the nationally representative data for the Somali region. The exertion made in decreasing under-five children’s undernutrition in the region is still high, and more effort is required to move forward the obstructions for advance diminishment. More research studies are, subsequently, required to inform policymakers to execute suitable mediation programs. To address these gaps, an all-inclusive cross-sectional analysis of the recent 2016 Ethiopian Demographic Health Survey (EDHS) was done, to explore the potential risk factors of undernutrition among under-five children in Somali region, Ethiopia.

## Methods

### Description of study design and area

A cross-sectional study design was used for this study. The study was conducted in Somali regional state which is located in the east and southeast of Ethiopia. According to the 2007 Census, the state’s population was 4,439,147 of which 1,970,363 were males. The urban residents of the region were 621,210 and its rural residents 3,817,937 [15].

### Data sources

The data for this study was extracted from the Ethiopian Demographic and Health Survey (EDHS) 2016. The Central Statistics Agency (CSA), the Ministry of Health (MOH), and the Ethiopian Public Health Institute together surveyed from January 18, 2016–June 27, 2016, where the United States Agency for International Development (USAID) funded the project. The authors have got permission from the ICF-DHS program to use the EDHS data and access it through https://www.dhsprogram.com/data/dataset_admin/login_main.cfm. The 2016 EDHS used a two-stage stratified sampling to select households. In the first stage, there were 645 enumeration areas (202 in urban and 443 in rural areas). 1339 (700 males and 639 females) under-five children from Somali region were considered for this study. This study considered live children age 0–59 months with anthropometry data in the analysis of determinants of nutritional status among children under age 5 in Somali region. Missing values in the 2016 EDHS dataset were not included in the analyses.

### Variables of the study

The subordinate factors for this consideration were the malnutrition status of under-5 year children (stunting, underweight and wasting). Children whose height-for-age Z-score is below minus two standard deviations (− 2SD) from the median of the reference population is considered as stunted. If the weight-for-age Z-score is below minus two standard deviations (− 2 SD) from the medium of reference population then the child is underweight. Children whose weight for height Z-score is below minus two standard deviations (−2SD) from the median of the reference population are considered as wasted [16]. Illustrative factors were chosen after conducting a point-by-point writing survey [16–21] additionally accessible with complete data in the EDHS, 2016 data set was included within the current investigation. Selected illustrative factors were divided into two levels which included socio-demographic-maternal and child-level variables. Socio demographic-maternal variables chosen were types of residence, household wealth index, mother’s educational level, mother’s body mass index (BMI), religion, and type of toilet facility, sex of household head and sources of drinking water. Child-level components were the sex of the child, child age, type of birth, number of living children and child size at birth. Malnutrition indicators were defined using the WHO child growth standards [22].

### Statistical data analysis

The data was extracted, edited, and analyzed by using SPSS version 23 for Windows. Descriptive statistics such as frequencies and proportions were used to summarize the distribution of selected background characteristics of the sample. Bivariate logistic regression was performed to analyze the crude odds ratio and a variable with a *P*-value of less than 0.25 was transported into multivariable binary logistic regression analyses to analyze adjusted odds ratio and to identify the determinants of undernutrition of children under 5 years old. The dependent variables for bivariate and multivariable logistic regression analyses were stunting, wasting and underweight. Finally, variables with *P*-values < 0.05 in the multivariable logistic regression model were taken as statistically significant.

## Results

In this study, a total of 1339 under-five children were included. In the Somali region, the prevalence of stunting, underweight and wasting were 27.4, 28.7 and 22.7%, respectively. Almost 16.1% of children were both stunted and underweight; the prevalence of underweight and wasting was 11.7%, the prevalence of stunted and wasted was 5.5%, and all three malnutrition conditions were only 4.7% children. Among the total participants, 555 children (41.4%) were 0–24 months old, 501 (37.4%) were 25–47 months old and 283 (21.1%) were 48–59 months old. From the total participants, male children constituted 700 of the sample population (52.3%), and nearly 1069 (79.8%) of the children were taken from rural areas while. The majority of the children (62.4%) were anemic; most children were born in single birth type (98.2%). The majority of children (56.5%) were born from overweight mothers, 24.6% of children were from normal-weight mothers and only 18.8% of children were from underweight mothers. Regarding the educational status of the household, 85.4% of households did not attend at all, 10.7% of households attended primary school and 3.8% of households attended secondary and higher education. The majority (63.8%) of the children were born at normal size, 32.3% were large and 24.6% of the children were born in small size at birth. More than half (63.8%) of the respondents had no toilet facilities; from the total participants, one thousand ninety-seven (81.9%) children were from the household who used unimproved water sources. The majority of children (75.8%) were born from poor wealth index families, 18.4% of children were from rich wealth index family and the rest 5.8% of children were from medium wealth index families. The number of children who lived in household members of 1–2, 3–4 and > 4 was 20.9, 30.4 and 48.7%, respectively. More than half of the respondents (67.8%) were male household-headed.

### Factors associated with stunting

According to the multivariable logistic regression analysis, age of the child, type of birth, toilet facility and anemia level was significantly associated with being stunted. The risk of being stunted was 1.94 times more likely among children that were aged 25–47 months as compared to those aged 0–24 months (AOR = 1.94; 95% CL: 1.41–2.69). The risk of being stunted was 1.66 times more likely among children that were aged 48–59 months as compared to those aged 0–24 months (AOR = 1.66; 95% CL: 1.12–2.46). The odds of stunting among children who were born in multiple-birth types were 2.86 times higher as compared to those born in single birth type (AOR = 2.86; 95% CL: 1.10–7.37). Children from the household who had not toilet facility were 1.45 times more likely to be stunted as compared to the children household who had a toilet facility (AOR = 1.45; 95% CL: 1.12–1.87). Being stunted of anemic children was 2.36 times more likely to be stunted as compared to non-anemic children (AOR = 2.36; 95% CL: 1.55–3.61) (Table 1).

**Table 1:** Bivariate and multivariable logistic regression of determinants associated with stunting on childhood under 5 years old in Somali Region, Ethiopia, EDHS 2016

Variables		Stunting	COR (95% CI)	AOR (95% CI)	***P***-value
	Yes	No			
**Age of child in months**					
0–24	115 (20.7%)	440 (79.3%)	1	1	
25–47	172 (34.4%)	328 (65.6%)	2.01 (1.52, 2.64)	1.94 (1.41, 2.69)***	< 0.000
48–59	80 (28.3%)	203 (71.7%)	1.51 (1.08, 2.10)	1.66 (1.12, 2.46)*	0.012
**Sex of child**					
Male	202 (28.9%)	497 (71.1%)	1	1	
Female	165 (25.8%)	474 (74.2%)	1.17 (0.92, 1.49)	0.77 (0.58, 1.02)	
**Place of residence**					
Urban	69 (25.7%)	200 (74.3%)	1	1	
Rural	298 (27.9%)	771 (72.1%)	1.12 (0.83, 1.52)	0.73 (0.45, 1.19)	
**Type of birth**					
Single birth	357 (27.2%)	957 (72.8%)	1	1	
Multiple birth	10 (41.7%)	14 (58.3%)	1.92 (0.84, 4.35)	2.86 (1.10, 7.37)*	0.031
**Mothers` BMI**					
Over weight	55 (21.8%)	197 (780.2%)	1	1	
Normal weight	213 (28.2%)	543 (71.8%)	1.41 (1.01, 1.97)	1.07 (0.73, 1.58)	
Under weight	99 (30.0%)	231 (70.0%)	1.54 (1.05, 2.25)	1.24 (0.80, 1.93)	
**Size of child at birth**					
Large	105 (24.2%)	328 (75.8%)	1	1	
Medium	162 (28.1%)	414 (71.9%)	1.22 (0.92, 1.63)	1.17 (0.85, 1.62)	
Small	100 (30.4%)	229 (69.6%)	1.36 (0.99, 1.88)	1.24 (0.86, 1.79)	
**Present of toilet facility**					
Yes	111 (22.9%)	374 (77.1%)	1	1	
No	256 (30.0%)	597 (70.0%)	1.73 (0.51, 1.03)	1.45 (1.12, 1.87)**	0.005
**Sex of household head**					
Male	259 (28.6%)	648 (71.4%)	1	1	
Female	108 (25.1%)	323 (74.9%)	0.84 (0.64, 1.09)	0.95 (0.71, 1.28)	
**Household wealth index combined**					
Poor	298 (29.4%)	717 (70.6%)	1	1	
Medium	14 (17.9%)	64 (82.1%)	0.53 (0.29, 0.95)	0.56 (0.28, 1.11)	
Rich	55 (22.4%)	190 (77.6%)	0.70 (0.50, 0.97)	0.78 (0.43, 1.40)	
**Number of living children**					
1–2	70 (25.1%)	209 (74.9%)	1	1	
3–4	109 (26.8%)	298 (73.2%)	1.09 (0.77, 1.55)	1.03 (0.68, 1.56)	
> 4	188 (28.8%)	464 (71.2%)	1.21 (0.88, 1.67)	1.01 (0.68, 1.49)	
**Sources of drinking water**					
Improved	62 (25.7%)	179 (74.3%)	1	1	
Unimproved	305 (27.8%)	792 (72.2%)	1.11 (0.81, 1.53)	0.82 (0.55, 1.23)	
**Anemia level**					
Non-anemic	34 (18.6%)	149 (81.4%)	1	1	
Anemic	284 (34.0%)	551 (66.0%)	2.26 (1.52, 3.37)	2.36 (1.55, 3.61)***	< 0.000

*AOR* adjusted odds ratio, *COR* crude odds ratio, *CI* confidence interval

^*^significant *p* value < 0.05

^**^significant *p* value < 0.01

^***^significant *p* value < 0.001, 1 = reference

### Factors associated with underweight

Results of the multivariable binary logistic regression model revealed that the age of a child, size of child at birth, and anemia level were significantly associated with underweight. The children who were 25–47 months aged groups were 1.67 times more likely to develop underweight as compared to those who were 0–24 months aged groups (AOR = 1.67; 95% CL: 1.22–2.28). The children who were 48–59 months aged groups were 1.70 times more likely to develop underweight as compared to those who were 0–24 months aged groups (AOR = 1.70; 95% CL: 1.17–2.46). Compared to children of a large size at birth, the odds of underweight among children in the small size at birth was 1.55 times higher (AOR = 1.55; 95% CL: 1.08–2.21). The anemic children were 1.65 times more likely to be underweight as compared to non-anemic children (AOR = 1.65; 95% CL: 1.12–2.43) (Table 2).

**Table 2:** Bivariate and multivariable logistic regression of risk factors associated with underweight on childhood less than 5 years in Somali Region, Ethiopia, EDHS 2016

Variables		Underweight	COR (95% CI)	AOR (95% CI)	***p***-value
	Yes	No			
**Age of child in months**					
0–24	124 (22.3%)	431 (77.7%)	1	1	
25–47	171 (34.1%)	330 (65.9%)	1.80 (1.37, 2.36)	1.67 (1.22, 2.28)***	0.001
48–59	89 (31.4%)	194 (68.6%)	1.60 (1.16, 2.20)	1.70 (1.17, 2.46)**	0.005
**Sex of child**					
Male	204 (29.1%)	496 (70.9%)	1	1	
Female	180 (28.2%)	459 (71.8%)	0.95 (0.76, 1.21)	0.89 (0.68, 1.16)	
**Place of residence**					
Urban	73 (27.0%)	197 (73.0%)	1	1	
Rural	311 (29.1%)	758 (70.9%)	1.11 (0.82, 1.49)	0.72 (0.45, 1.17)	
**Type of birth**					
Single birth	376 (28.6%)	939 (71.4%)	1	1	
Multiple birth	8 (33.3%)	16 (66.7%)	1.25 (0.53, 2.94)	1.26 (0.48, 3.33)	
**Mothers` BMI**					
Over weight	60 (23.8%)	192 (76.2%)	1	1	
Normal weight	215 (28.4%)	542 (71.6%)	1.27 (0.91, 1.77)	1.10 (0.75, 1.61)	
Under weight	109 (33.0%)	221 (67.0%)	1.58 (1.09, 2.28)	1.29 (084, 1.98)	
**Size of child at birth**					
Large	109 (25.2%)	324 (74.8%)	1	1	
Medium	160 (27.7%)	417 (72.3%)	1.14 (0.86, 1.51)	1.17 (0.85, 1.61)	
Small	115 (35.0%)	214 (65.0%)	1.60 (1.71, 2.19)	1.55 (1.08, 2.21)*	0.017
**Present of toilet facility**					
Yes	259 (30.3%)	595 (69.7%)	1	1	
No	125 (25.8%)	360 (74.2%)	1.25 (0.98, 1.61)	0.84 (0.60, 1.18)	
**Sex of household head**					
Male	265 (29.2%)	642 (70.8%)	1	1	
Female	119 (27.5%)	313 (72.5%)	0.92 (0.71, 1.19)	0.98 (0.73, 1.31)	
**Household wealth index combined**					
Poor	302 (29.8%)	713 (70.2%)	1	1	
Medium	24 (30.8%)	54 (69.2%)	1.05 (0.64, 1.73)	0.96 (0.53,1.74)	
Rich	58 (23.6%)	188 (76.4%)	0.73 (0.53, 1.01)	0.59 (0.33, 1.06)	
**Number of living children**					
1–2	73 (26.1%)	207 (73.9%)	1	1	
3–4	122 (30.0%)	285 (70.0%)	1.21 (0.86, 1.71)	1.16 (0.78, 1.75)	
> 4	189 (29.0%)	463 (71.0%)	1.16 (0.84, 1.59)	1.11 (0.75, 1.63)	
**Sources of drinking water**					
Improved	69 (28.5%)	173 (71.5%)	1	1	
Unimproved	315 (28.7%)	782 (71.3%)	1.01 (0.74, 1.37)	0.89 (0.61, 1.32)	
**Anemia level**					
Non-anemic	46 (25.0%)	280 (70.9%)	1	1	
Anemic	287 (34.4%)	548 (65.6%)	1.57 (1.09, 2.26)	1.65 (1.12, 2.43)*	0.011

*AOR* adjusted odds ratio, *COR* crude odds ratio, *CI* confidence interval

^*^significant *p* value < 0.05

^**^significant *p* value < 0.01

***significant *p* value < 0.001, 1 = reference

### Factors associated with wasting

Based on multivariable logistic regression analysis, sex of the child, Mothers’ BMI, size of child at birth and sources of drinking water were significantly associated with wasting. The results of the adjusted odds ratio showed that male children were 0.35 times less likely to be wasting compared to female children (AOR = 0.65; 95% CL: 0.48–0.88). The risk of being wasted among children who were born from underweight mothers was 1.64 times higher compared to those born from overweight mothers (AOR = 1.64; 95% CL: 1.03–2.64). The children who had small size at birth were 1.58 times more likely to be wasting compared to those who had large size at birth (AOR = 1.58; 95% CL: 1.07–2.31). Children whose household used unimproved water were 1.66 times more likely to be wasted as compared to the children household used improved water (AOR = 1.66; 95% CL: 1.44–3.98) (Table 3).

**Table 3:** Bivariate and multivariate logistic regression of risk factors associated with wasting on childhood less than 5 years old in Somali Region, Ethiopia

Variables		Wasting	COR (95% CI)	AOR (95% CI)	***p***-value
	Yes	No			
**Age of child in months**					
0–24	120 (21.6%)	435 (78.4%)	1	1	
25–47	117 (23.4%)	384 (76.6%)	1.10 (0.83, 1.48)	1.07 (0.76, 1.49)	
48–59	67 (23.7%)	216 (76.3%)	1.12 (0.80, 1.58)	1.14 (0.76, 1.72)	
**Sex of child**					
Male	181 (25.9%)	519 (74.1%)	1	1	
Female	123 (19.2%)	516 (80.8%)	0.68 (0.53, 0.89)	0.65 (0.48, 0.88)**	0.005
**Place of residence**					
Urban	65 (24.1%)	205 (75.9%)	1	1	
Rural	239 (22.4%)	830 (77.6%)	0.91 (0.66, 1.24)	0.79 (0.48, 1.31)	
**Type of birth**					
Single birth	300 (22.8%)	1015 (77.2%)	1	1	
Multiple birth	4 (16.7%)	20 (83.3%)	0.68 (0.23, 1.99)	0.61 (0.17, 2.15)	
**Mothers` BMI**					
Over weight	47 (18.7%)	205 (81.3%)	1	1	
Normal weight	168 (22.2%)	589 (77.8%)	1.24 (0.87, 1.78)	1.38 (0.91, 2.11)	
Under weight	89 (27.0%)	241 (73.0%)	1.61 (1.08, 2.40)	1.64 (1.03, 2.64)*	0.038
**Size of child at birth**					
Large	90 (20.8%)	343 (79.2%)	1	1	
Medium	130 (22.5%)	447 (77.5%)	1.11 (0.82, 1.50)	1.19 (0.84, 1.67)	
Small	84 (25.5%)	245 (74.5%)	1.13 (0.93, 1.84)	1.58 (1.07, 2.31)*	0.021
**Present of toilet facility**					
Yes	191 (22.4%)	663 (77.6%)	1	1	
No	113 (23.3%)	372 (76.7%)	1.05 (0.81, 1.37)	1.06 (0.74, 1.53)	
**Sex of household head**					
Male	211 (23.3%)	696 (76.7%)	1	1	
Female	93 (21.5%)	339 (78.5%)	0.91 (0.69, 1.19)	0.86 (0.63, 2.16)	
**Household wealth index combined**					
Poor	229 (22.6%)	786 (77.4%)	1	1	
Medium	20 (25.6%)	58 (74.4%)	1.18 (0.70, 2.01)	1.17 (0.63, 2.16)	
Rich	55 (22.4%)	191 (77.6%)	0.99 (0.71, 1.38)	0.93 ()0.51, 1.69	
**Number of living children**					
1–2	60 (21.4%)	220 (78.6%)	1	1	
3–4	93 (22.9%)	314 (77.1%)	1.09 (0.75, 1.57)	1.02 (0.66, 1.58)	
> 4	151 (23.2%)	501 (76.8%)	1.11 (0.79, 1.55)	102 (0.68, 1.55)	
**Sources of drinking** water					
Improved	67 (27.7%)	175 (72.3%)	1	1	
Unimproved	237 (21.6%)	860 (78.4%)	1.72 (1.52, 2.99)	1.66 (1.44, 3.98)*	0.041
**Anemia level**					
Non-anemic	44 (23.9%)	140 (76.1%)	1	1	
Anemic	209 (25.0%)	626 ()75.0%	1.06 (0.73, 1.54)	1.09 (0.73, 1.61)	

*AOR* adjusted odds ratio, *COR* crude odds ratio, *CI* confidence interval

^*^significant *p* value < 0.05

^**^significant *p* value < 0.01

***significant *p* value < 0.001, 1 = reference

## Discussion

In this study, the prevalence of stunting in the present study was 27.4%. This prevalence is lower than the previous studies conducted in in Ethiopia (38.3%) [16], in Shabelle zone, in Shinille District (33.4%) [23], in Tigray region 39.1% [24], Takusa District (36.5%) [25], in rural Ethiopia (41.2%) [26], in east Gojjam Zone (44.7%) [27], in Tigray region (46.9%) [11] and in Hidabu Abote District (47.6%) [28], but it was higher than the study conducted in the Bure Town of West Gojjam Zone (24.9%) [29]. Similar studies conducted in Nairobi Peri-Urban Slum and Nigeria reported a higher prevalence of stunting (30.2%) [30] and (47.6%) [31], respectively. The variation might be due to socioeconomic, geographical characteristics of the study area, cultural differences in dietary habits and care practices.

In the present study, the prevalence of underweight was 28.7%. The prevalence of underweight in this finding is higher than the studies conducted in Ethiopia (23.3%) [16], in Tigray (23.9%) [24], Dale district (19%) [32], Takusa District (19.5%) [25], in rural Ethiopia (27%) [26], in east Gojjam Zone (15.30%) [27] and the Bure Town of West Gojjam Zone (14.30%) [29], but it is lower than the study conducted in Hidabu Abote District (30.9%) [28]. The lower prevalence of underweight was reported in Nairobi Peri-Urban Slum (14.9%) [30] and Nigeria (25.6%) [31]. This could be because the households lack knowledge, attitude and practices (KAP) on how to feed their children and themselves [33].

The prevalence of wasting was 22.7%. The prevalence of wasting in the present study is higher compared to the study conducted in Ethiopia (10.1%) [16], in Haramaya district (10.7%) [34], in Tigray region (11.6%) [11], in Dale district (14%) (32), in Pakistan (10.7%) [33], Nairobi Peri-Urban slum (4.5%) [30], and (16.7%) [28], in the Bure Town of West Gojjam Zone (11.1%) [29] and in east Gojjam Zone (10%) [27]. In general, this high prevalence of wasting could be due to repeated attacks of drought and famine in the study area.

According to the multivariable logistic regression analysis, age of the child, type of birth, toilet facility and anemia level was significantly associated with being stunted. The risk of being stunted was 1.94 and 1.66 times more likely among children that were aged 25–47 and 48–59 as compared to those aged 0–24 months, respectively. This finding is in line with the studies conducted in Ethiopia [16], in Haramaya district [34], in Pakistan [33], in Amhara region [35] and Kilimanjaro Region, Tanzania [36]. This could be because younger children are more likely to receive more attention and feeding effort from their parents as compared to older children [37]. It could also be due to the inappropriate and late introduction of low nutritional quality supplementary food [38]. The risk of being stunted among children who were born in multiple-birth types was 2.86 times higher as compared to those born in single birth types. This finding is consistent with the study carried out in Tigray region [24]. The reason behind might be in multiple birth types, there could be food competition between children and it leads to malnutrition; and the mothers’ breast may not produce enough milk for both children. Children from the household who had not toilet facility were 1.45 times more likely to be stunted compared to the children household that had a toilet facility. This result is in contradiction to the studies conducted in Tigray region [24] and in Bule Hora district, South Ethiopia [39]. This might be because lack of toilet facility is the main cause for intestinal parasites and microorganisms which leads to loss of appetite leading to poor nutritional status; this might repeated infection causes depressed immunity and making the severity and duration of disease more severe contributing to the poor nutritional status of the children. Being stunted of anemic children was 2.36 times more likely to be stunted as compared to non-anemic children. This finding has supported the study conducted in Ethiopia [16]. Undernourished children are more suffered from inadequate bioavailability of micronutrients such as iron, B12 and folate in their body which are important for the formation of blood cells. Therefore, those children who are undernourished cannot form adequate blood cells as much as required; consequently the this leads to the development of nutritional deficiency anemia which is common especially in developing countries [40].

The children who were 25–47 and 48–59 aged groups were 1.67 and 1.70 times more likely to develop underweight as compared to those who were 0–24 aged groups. This finding is supported by the study conducted in Ethiopia [16], but in contradiction to the study conducted in Tigray region [24] and in Pakistan [33]. This might be due to the fact that as children grow older, they may have less access to attention and not provide sufficient food by their families. It might be due to a large portion of guardians in rural areas are ignoring to meet their children’s optimal food requirements like the age of the child increases [41]. Compared to children of a large size at birth, the odds of underweight among children in the small size at birth was 1.55 times higher. This study is in line with the study conducted in Tigray region [24] and in Pakistan [33]. Children with low birth sizes are often disadvantaged in terms of physical growth and are also more vulnerable to infections compared to children born with normal birth sizes [42]. The anemic children were 1.65 times more likely to be underweight as compared to non-anemic children. This might be due to the fact that the communities in the study area consume cereals that have low iron content [43].

The results of the adjusted odds ratio showed that the sex of the child was significantly associated with wasting. Wasting is a relatively short-term condition, which means that an individual child can be affected more than once in a year [44]. Male children were 0.35 times less likely to be wasting compared to female children. This finding is consistent with finding in Tigray region [24], but previous studies indicated that boys had a significantly worse nutritional status than girls [24]. This could probably be due to the value and cultural preferences placed on the male child. As such, they are likely to be better fed as compared to the girl child. This has also been shown in other Sub-Saharan African countries [45]. The risk of being wasting among children who were born from underweight mothers was 1.64 times higher compared to those born from overweight mothers. This study is in line with findings in Pakistan [33], Ethiopia [16], India [46] and Vietnam [47]. This finding is also similar to other previously conducted studies [34, 48]. This could be explained by the presence of an intergenerational link between maternal and child nutrition means a small mother will have small babies who in turn grow to become small mothers [49]. Maternal BMI is also an important determinant of child under-nutrition and is influenced by maternal nutrition, to improve child growth, proper nutrition is essential for the mothers during the prenatal and postnatal period. Healthier mothers have less risk of having undernourished children [18]. The children who had small sizes at birth were 1.58 times more likely to be wasting compared to those who had large sizes at birth. This result is supported by the study conducted in Tigray region [24]. Children whose households used unimproved water were 1.66 times more likely to be wasting as compared to the children household used improved water. This finding is supported by finding in Haramaya District, Eastern Ethiopia [34]. This might be due to the fact that impure water is a vehicle for intestinal parasites which leads to loss of appetite leading to poor nutritional status; this might repeated infection causes depressed immunity and making the severity and duration of disease more severe contributing to the poor nutritional status of the children.

## Conclusion

This study revealed individual- and community-level factors that determined childhood malnutrition in Somali region children. Among the factors considered in this study, age of the child in months, type of birth, anemia level, size of child at birth, sex of the child, mothers’ BMI and sources of drinking water were significantly associated with stunting, underweight and wasting. The authors concluded that under-nutrition among under-five children was one of the public health problems in the study area. Thus, interventions should be focused on 25–59 months age of children, multiple birth type, anemic children, and small size of child at birth, female children, underweight mothers and improving access to improved drinking water to get better health care, to enhance the child’s nutritional status, and reduce child mortality more rapidly.

## Data Availability

Data will be available upon request from the corresponding author.

## Abbreviations

AOR: Adjusted odds ratio
BMI: Body mass index
COR: Crude odds ratio
CSA: Central Statistical Agency
SPSS: Statistical Package for Social Science
DHS: Demographic and Health Surveys
EDHS: Ethiopian Demographic and Health Survey
